# Mutations in Selected ABA-Related Genes Reduce Level of *Arabidopsis thaliana* Susceptibility to the Beet Cyst Nematode *Heterodera schachtii*

**DOI:** 10.3390/plants12122299

**Published:** 2023-06-13

**Authors:** Elżbieta Różańska, Tomasz Krępski, Anita Wiśniewska

**Affiliations:** 1 Department of Botany, Institute of Biology, Warsaw University of Life Sciences—SGGW, Nowoursynowska 159, 02-776 Warsaw, Poland; 2 Department of Plant Physiology, Institute of Biology, Warsaw University of Life Sciences—SGGW, Nowoursynowska 159, 02-776 Warsaw, Poland

**Keywords:** *Arabidopsis thaliana*, cyst nematode, *Heterodera schachtii*, abscisic acid, plant susceptibility

## Abstract

*Heterodera schachtii* is a common parasite of many important crops such as beets and *Brassicaceae* (oilseed rape, cabbage or mustard). *Arabidopsis thaliana* is a model plant also used for studying defence responses to pathogens or pest infections. Defence responses of plants are often regulated and fine-tuned by stress phytohormones: salicylic acid (SA), jasmonic acid (JA), ethylene (Et) and abscisic acid (ABA), of which the role of ABA in these responses is the least examined. The aim of this study was to show, if and which genes related to ABA turnover can be modulated during the development of nematode-induced feeding sites in *A. thaliana* roots. To answer the question, we performed infection tests on wild type and ABA mutant roots and analysed the expression levels of selected ABA-related genes (*ABI1*, *ABI2*, *ABI5*, *PYL5*, *PYL6*, *CYP707A1* and *CYP707A4*) at the early stage of root infection. Our results show that the expression of *ABI2*, *ABI5* (ABA signalling) and *CYP707A4* (ABA metabolism) genes was upregulated in feeding sites at 4 dpi, whereas the level of expression of *PYL5* and *PYL6* (ABA receptors) genes was decreased. Mutations in *ABI1*, *ABI2*, *ABI5*, *CYP707A1* or *CYP707A4* genes led to a decrease of *A. thaliana* susceptibility verbalised as the number of fully developed females, whereas mutations in *PYL5* or *PYL6* genes did not influence the number of females of the nematode. Based on the results, it can be concluded that the modifications of analysed ABA-related gene expression are required for the proper development of nematodes; however, further in-depth analyses are required.

## 1. Introduction

Plant parasitic nematodes are small roundworms which attack plant roots and cause serious crop yield losses worldwide [1]. It was estimated that damage caused by them exceeds 80 billion USD yearly [2]. Sedentary root endoparasitic (root-knot and cyst forming) nematodes induce and maintain specialised feeding sites that are composed of modified plant root cells [3]. The most damaging species of cyst nematodes are potato cyst nematodes (*Globodera pallida* and *G*. *rostochiensis*), soybean cyst nematodes (*Heterodera glycines*) and cereal cyst nematodes (*H*. *avenae* and *H*. *filipjevi*) [4].

The second stage juveniles (J2) of cyst nematodes, after hatching from the egg, migrate in the soil and locate roots of their host. They invade the root and migrate intracellularly to find the proper plant cell which can be used to become the initial syncytial cell (ISC). The J2 inserts its stylet into selected ISCs and injects secretions originating from its pharyngeal glands, which induce the development of a nematode feeding site called a syncytium [5]. The syncytium is strongly hypertrophied and a multinucleate cell, formed by fusion of protoplasts of adjacent strongly enlarged parenchymatic cells through the formation of partial cell wall dissolutions. The juveniles of the nematode remain attached to the feeding site and withdraw nutrients. Meanwhile, they undergo three moults to the adult stage (females or males). After fertilization and oviposition, the females die and their bodies form cysts protecting the eggs [5].

Sedentary endoparasites are able to manipulate plant cell metabolism by injecting their secretions containing effectors. Nematode effectors can be proteins and other chemical compounds, which suppress plant defence responses during root invasion, J2s migration and feeding site establishment and maintenance. Effectors secreted into the plant cell also trigger transcriptional and metabolic reprogramming, plant protein degradation and stress response. Effectors of parasitic nematodes are produced in the pharyngeal gland cells and are secreted through the hollow stylet [6].

Plant phytohormones play important roles in the regulation of developmental processes and signal transduction pathways involved in plant responses to a wide range of biotic and abiotic stresses. The defence response of plants to pathogen infection often depends on the action of stress phytohormones: salicylic (SA), jasmonic (JA) and abscisic (ABA) acids and ethylene (Eth). The role of SA, JA or Eth in the regulation of plant defence responses to bacteria or fungi is well characterized. SA signal transduction appears to play the pivotal role during biotrophic pathogen infection whereas plant responses to necrotrophic pathogens rely on JA-related pathways [7]. In contrast to its generally protective role in abiotic stress, ABA may promote either susceptibility or resistance to pathogens, depending on the pathogens and their modes of infection (reviewed in [8,9]). However, the knowledge about the role of ABA in plant responses to nematode infection is very restricted.

The synthesis of ABA from C_40_ carotenoids involves several enzymatic steps, employing enzymes encoded by *ZEAXANTHIN EPOXIDASE* (*ZEP, ABA1*), *9-CIS-EPOXYCAROTENOID DIOXYGENASE* (*NCED*) and *ABSCISIC ALDEHYDE OXIDASE* (*AAO3*) genes [10]. The production of xanthoxin catalysed by NCED is thought to be the key regulatory step in ABA biosynthesis. Xanthoxin is next converted into abscisic aldehyde by a short-chain alcohol dehydrogenase/reductase (SDR1), encoded by the *AtABA2* gene, and next, abscisic aldehyde is oxidized by AAO into ABA [11,12].

In mutational screening, five ABA-insensitive mutants have been identified and designated as *abi1* up to *abi5*. The corresponding *ABI1* and *ABI2* genes encode PP2Cs (TYPE 2C PROTEIN PHOSPHATASES), while *ABI3*, *ABI4* and *ABI5* encode transcription factors involved in ABA signalling pathway. As a result of detailed research, 14 members of (StAR)-related lipid-transfer (START) domain/major birch pollen allergen (Bet v 1) superfamily of proteins were proposed as ABA receptor candidates. These 14 proteins were named PYRABACTIN RESISTANCE 1 (PYR1) and PYR1-like 1–13 (PYL1-PYL13) or REGULATORY COMPONENT OF ABA RECEPTOR (RCAR1-RCAR14) [12]. During signal transduction in the absence of ABA, SnRK2s (Snf1-RELATED PROTEIN KINASE 2) are inactivated by PP2Cs. When ABA binds to PYL proteins, PYLs interact with PP2Cs and inhibit their activity, and SnRK2s are activated by the release from negative regulation of PP2Cs. Activated SnRK2 kinases phosphorylate bZIP transcription factors, which upregulate ABA-responsive genes [12].

ABA catabolism encompasses conversion of ABA to 8′-hydroxy ABA by CYP707A family cytochrome P450 monooxygenase and next 8′-hydroxy ABA is isomerised spontaneously to phaseic acid (PA). The isomerisation is reversible. PA can be subsequently converted to dihydrophaseic acid (DPA) by PA reductase (PAR)—ABH2 (ABA HYPERSENSITIVE 2). Catabolism of ABA is impaired in the *abh2* mutant, which contains very low amounts of DPA and higher levels of PA, while the ABA level is comparable to the levels in the wild type plants [13].

ABA is considered as a hormone primarily involved in plant responses to abiotic stress (in particular drought and salinity stress). ABA can also play a pivotal role in plant immunity. It was shown that ABA can interact antagonistically with SA and JA/Eth. Investigation on plant defence response, including ABA biosynthesis mutants and ABA signalling genes, showed a negative role of ABA in plant immunity to bacterial and fungal infections. It was also shown that fungal pathogens are able to produce their own ABA [14]. However, examples for ABA-induced resistance also exist. The positive effects of ABA were shown in plant responses to *necrotrophic fungal* pathogens [9]. The ABA treatment of potato plants decreased the production of egg masses by root-knot nematode *M*. *incognita* [15]. The role of ABA was also evidenced in the systemic-induced defence of rice to the migratory root-knot nematode *Hirschmanniella oryzae*. Foliar ABA application suppressed rice basal immunity and indicated an antagonistic interaction between ABA and SA/JA/Eth pathways [14].

*A. thaliana* is a good model host to study plant–parasitic nematode interactions, including beet cyst nematode *H. schachtii* [16]. Kammerhofer et al. [17] established that *H*. *schachtii* infection induces changes in concentrations of endogenous phytohormones in *A. thaliana* roots. The content of JA and the immediate Eth precursor, ACC, was strongly increased, whereas the content of ABA and gibberellin 4 GA4 was reduced 24 h after inoculation compared with noninfected roots. Changes in concentrations of these hormones may modulate hormone-based defence and signal transduction. The concentrations of SA, IAA and active cytokinins were unchanged at this time point [17].

So far, the role of ABA was confirmed mainly in plant responses to abiotic stresses but the knowledge about its function in defence responses to nematode infection is limited. Therefore, the goal of this work was to analyse whether mutations in selected ABA-related genes can influence the root infection and *H. schachtii* development.

## 2. Results

### 2.1. Analysis of Changes in A. thaliana Gene Expression at Early Stage of Nematode Parasitism

**Figure 1:**
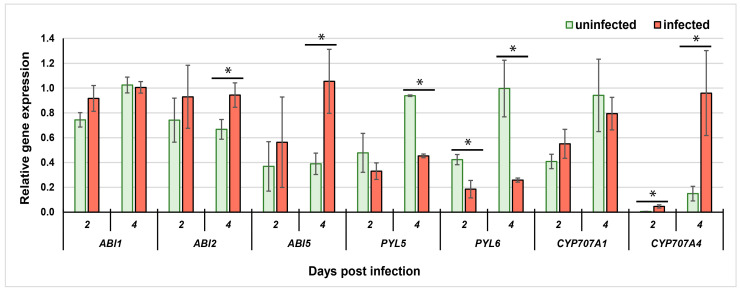
The relative expression levels of *ABI1*, *ABI2*, *ABI5*, *PYL5*, *PYL6*, *CYP707A1* and *CYP707A4* genes in uninfected control WT *A. thaliana* plants and syncytia induced by *H. schachtii* in WT roots at 2 and 4 dpi. The bars show mean values ±standard deviation. Data were obtained from three independent biological replicates. Asterisks indicate means, which are significantly different (*) at *p* < 0.05 according to ANOVA and a post hoc Fisher’s least significant difference (LSD) test.

To answer whether ABA plays a role in *A. thaliana*–*H. schachtii* interaction, we selected several genes implicated in ABA catabolism and signal transduction for detailed analysis. *ABI1*, *ABI2*, *ABI5*, *PYL5* and *PYL6* are involved in ABA signalling. There was no difference in *ABI1* gene transcript level in infected roots in comparison to uninfected roots, neither at 2 dpi nor at 4 dpi (Figure 1). The *ABI2* gene expression level did not differ between uninfected and infected roots at 2 dpi, but infected roots showed a higher relative *ABI2* gene expression than uninfected roots at 4 dpi (Figure 1). Similarly, the *ABI5* gene expression was not activated at 2 dpi, but up-regulation of this gene took place in infected roots at 4 dpi (Figure 1). The expression levels of genes encoding ABA receptors (*PYL5* and *PYL6*) in infected roots were reduced at 2 and 4 dpi for *PYL6*, but only at 4 dpi for *PYL5* when compared to uninfected roots (Figure 1). *CYP707A1* and *CYP707A4* participate in ABA metabolism. *CYP707A1* transcript accumulation levels did not change between infected and uninfected roots neither at 2 dpi nor 4 dpi. However, a rise of *CYP707A4* transcripts levels was found in samples collected at 2 and 4 dpi from infected roots when compared to uninfected roots (Figure 1).

### 2.2. Endogenous ABA Concentrations in Arabidopsis Mutants

**Figure 2:**
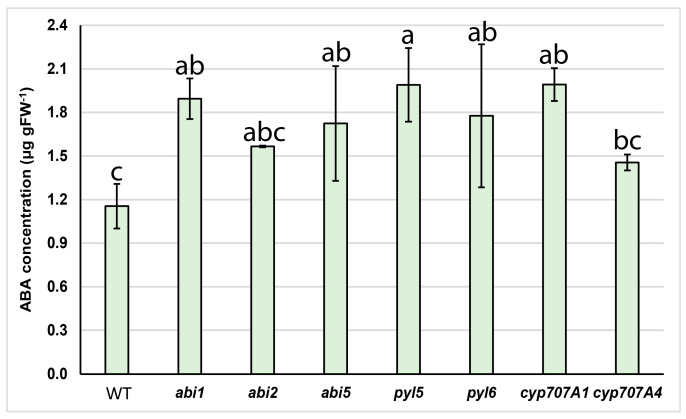
ABA concentrations in *abi1*, *abi2*, *abi5*, *pyl5*, *pyl6*, *cyp707A1* and *cyp707A4* mutants and wild type (WT) plants. ABA was measured by ELISA method. Values shown are means of four independent biological replicates ± standard deviation. Different letters on bars indicate statistically significant differences according to one−way ANOVA (*p* < 0.05) and a post hoc Fisher’s least significant difference (LSD) test). gFW, gram fresh weight.

In view of the fact that several mutants used in this work have not been previously described in the literature, we first estimated the amount of ABA in mutants and compared it with wild type plants grown under non-stress conditions. The statistically significant increase of ABA content was found in *abi1*, *abi5*, *pyl5*, *pyl6* and *cyp707A1* mutants compared to the wild type plant (Figure 2).

### 2.3. Susceptibility Level of Arabidopsis Mutants to *H. schachtii*

**Figure 3:**
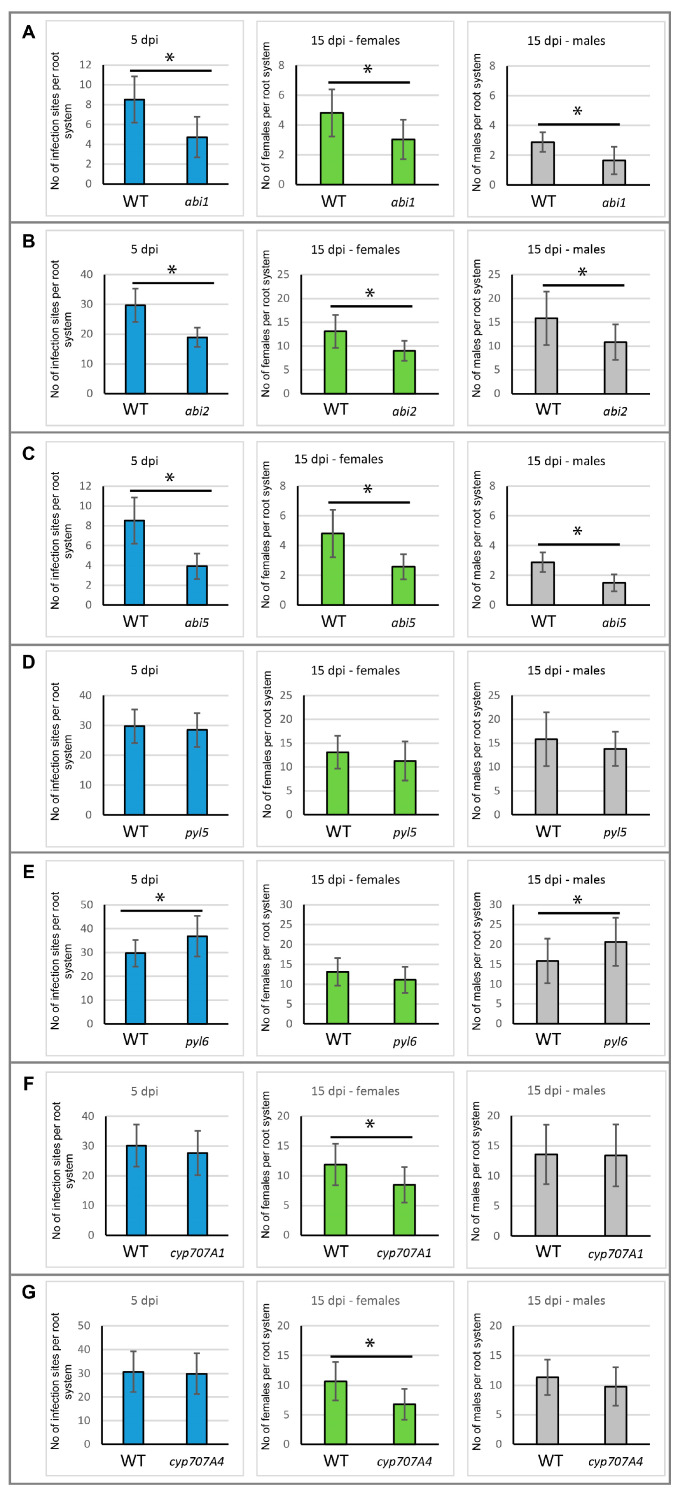
Development of *H. schachtii* on wild type (WT) and ABA-related mutants (*abi1* (**A**), *abi2* (**B**), *abi5* (**C**), *pyl5* (**D**), *pyl6* (**E**), *cyp707A1* (**F**) and *cyp707A4* (**G**)). Infection sites were counted at 5 dpi, and developed females and males at 15 dpi of WT and mutant roots. The bars show mean values ± standard deviation. Data obtained from at least nine independent biological replicates (n > 45). Asterisks indicate means, which are significantly different at *p*  <  0.05 according to ANOVA and a post hoc Fisher’s least significant difference test (LSD).

The commonly accepted parameter depicting plant susceptibility to parasitic nematodes is the average number of females developed on roots at 15 dpi [16]. In some instances, also the number of infection sites at 5 dpi and males at 15 dpi are counted and compared as additional indicators. For analysed *aba-insensitive* mutants (*abi1*, *abi2* and *abi5*), a reduction in the number of infection sites at 5 dpi, as well as the number of developed females and males, were observed (Figure 3A–C). For the *abi1* mutant, the decrease of infection sites number to 58%, number of females to 63% and number of males to 57% were shown (Figure 3A). For the *abi2* mutant, the infection sites number decreased to 64%, the number of females to 69% and males to 68% (Figure 3B). For the *abi5* mutant, the number of infection sites decreased to 48%, number of females to 54% and number of males to 57% when compared to the wild type plant (Figure 3C). Mutations in *ABI1*, *ABI2* and *ABI5* genes involved in ABA signalling always decreased susceptibility of *Arabidopsis* to *H. schachtii* infection.

In the case of the *pyl5* mutant, no differences in the number of infections sites at 5 dpi nor the number of nematodes at 15 dpi were found (Figure 3D). Similarly, in the *pyl6* mutant, no differences in the number of females was observed at 15 dpi, but the number of infection sites at 5 dpi was increased by 24% (*p* < 0.05), which probably led later to an increase in the number of males developed at 15 dpi by 30% (*p* < 0.05) (Figure 3E).

The average number of females developed at 15 dpi was decreased in the *cyp707A1* mutant line to 71%, whereas the number of infection sites and males did not show differences with the wild type plant (Figure 3F). Similarly, the average number of females developed on the *cyp707A4* mutant was decreased to 63% in comparison to the wild type plants, while the average number of infection sites at 5 dpi and males at 15 dpi was not altered by this mutation (Figure 3G).

## 3. Discussion

During evolution, plants developed the resistance mechanism against pathogens based on resistance (R) genes. However, pathogens have acquired the ability to break plant defence responses via evolution of so-called effectors [18]. To date, a few plant nematode resistance *R* genes have been identified and cloned. However, the transfer of *R* genes from donors to cultivars by means of crossing or genetic transformation faces many difficulties, where the most important is a lack of satisfactory effect of the *R* gene in a new genetic background [18]. After the discovery decades ago that a mutation in the *Mlo* gene led to the acquisition of powdery mildew resistance in barley, the approach of scientists and breeders slowly changed and an identification and functional analysis of potential susceptibility genes (*S* genes) became one of the main goals for new pathogen control strategies [19]. The function of *S* genes could be disrupted using site-specific mutagenesis based on nucleases technology of genome engineering. In this work, we analysed the influence of mutations in seven selected genes taking part in ABA signalling and turnover. Mutation in five of them showed the decrease of *Arabidopsis* susceptibility to cyst nematode infection. These five genes seem to play crucial roles in the regulation of plant response to beet cyst nematode infection, which facilitates proper development of the parasite.

Plant parasitic nematodes evolved sophisticated mechanisms modulating plant defence that facilitate the development and functioning of their feeding sites. Two main phases can be distinguished during parasitism: the first is a migration and syncytium induction and the second is syncytium development and maintenance. Most research concentrates on the second phase; however, according to the current knowledge, at the later phase when the syncytia are fully developed, defence mechanisms are suppressed in roots of susceptible plants. Similarly, so far, many studies have been focused on stress hormones, mainly SA and JA, while little is known about the involvement of ABA [20], especially during the first stage of plant–nematode interaction. Concentration of ABA was quantified in *A. thaliana* roots infected with *H. schachtii* by Kammerhofer et al. [17] and hormone reduction was observed at 1 dpi. Thus, in our work, we focus on genes participating in ABA catabolism and signal transduction.

The preliminary selection of genes of interest was based on gene expression analyses in *H. schachtii*-induced syncytia provided by Szakasits et al. [21]. Their data show differences in levels of gene expression between uninfected root segments and pooled 5/15 dpi syncytia. In our work, we focused on and analysed expression levels of selected genes during the early stage of plant infection in 2 and 4 dpi syncytia to corelate it with decreased ABA concentration at 1 dpi, showed by Kammerhofer et al. [17]. An especially strong induction of transcription of the *CYP707A4* gene was shown in 4 dpi syncytia (Figure 1). *CYP707A* genes are responsible for the conversion of ABA to 8′-hydroxy ABA and its spontaneous isomerisation to PA [13]. The increase of CYP707A4 could, therefore, be responsible for the decrease of ABA concentration 24 h after root infection, as shown by Kammerhofer et al. [17]. Concluding, mutation in ABA catabolism genes analysed herein did not influence infection of plants, as the number of infection sites on mutants and wild type roots were similar at 5 dpi despite the higher levels of their gene transcription.

The decreased number of infection sites was found in *abi1* (PP2C), *abi2* (PP2C) and *abi5* (transcription factor) signalling mutants, where an elevated expression of *ABI2* and *ABI5* genes was shown (Figure 1). In the case of the *PYL6* gene, encoding ABA receptor, the number of infection sites at 5 dpi was higher and the level of *PYL6* transcription was lower at 2 and 4 dpi than in wild type roots. Nonetheless, both *pyl 5* and *pyl 6* receptor mutants did not show significant differences in susceptibility levels to beet cyst nematode, presented as number of females.

Based on results obtained in this work, we can state that the action of five examined genes involved in ABA catabolism (*CYP707A1* and *CYP707A4)* or signalling (*ABI1*, *ABI2* and *ABI5*) seems to be required for proper syncytium formation and females of nematode development.

The regulation mechanism of ABA levels in plant cells is very complicated. In addition, individual genes involved in the ABA turnover belong to gene families, and functional redundancy by other genes from a given family cannot be ruled out. However, a clear phenotype of decreased susceptibility to beet cyst nematode (expressed as a number of developed females at 15 dpi) was shown in this work. It was also shown that ABA concentrations are regulated through a constant balancing between its synthesis and catabolism, including feedback induction of catabolism [22]. To obtain a more complete image, further detailed analyses such as ABA and its derivative concentration measurements in roots of mutants during infection, or analysis of double mutants or transgenic lines with overexpression of the studied genes, have to be carried out.

## 4. Materials and Methods

### 4.1. Plant Material and Growth Conditions

Wild type *Arabidopsis thaliana* ecotype Col-0 and T-DNA mutants (Table 1) were used in the experiments. Seeds of all plants were obtained from the Nottingham Arabidopsis Stock Centre (University of Nottingham, UK) and homozygous lines were selected by PCR-based genotyping according to the SIGnAL (Salk Institute Genomic Analysis Laboratory, USA) procedure, using the gene specific primers listed in Table 2 and T-DNA specific primer: SALK_LBb1.3; ATTTTGCCGATTTCGGAAC, Spm32; TACGAATAAGAGCGTCCATTTTAGAGTGA, or SAIL_LB3; TAGCATCTGAATTTCATAACCAATCTCGATACAC). Homozygous lines were selected for all mutants, except for the *CYP707A4* gene mutant. Despite screening about a hundred plants, only wild type or heterozygotes were found in the analysed population. This may indicate the lethality of the mutation in a homozygous form.

*A. thaliana* seeds were surface-sterilized for 2 min in 95% (*v*/*v*) ethanol and next in 5% (*v*/*v*) sodium hypochlorite (Sigma-Aldrich, St. Louis, MO, USA) for 8 min, washed 3 times in dH_2_O and placed on modified ‘KNOP’ medium, 5 plants per Ø 90 mm plate [16]. Seeds were stratified at 4 °C in dark for 2 days after being placed on the medium. Plants were cultivated under a 12 h day/12 h night photoperiod (125 µmol m^−2^ s^−1^) at 21 °C.

### 4.2. Nematode Culture, Infection Assays and Measurements

Cysts and infective second-stage juveniles of *Heterodera schachtii* Schmidt were prepared as described previously [27]. In brief, *A. thaliana* mutant plants and WT (Col-0) were inoculated with 70 infective second-stage juveniles per plant under sterile conditions. The number of infection sites (at 5 dpi) and the number of females and males (at 15 dpi) were counted per plant. Homozygous mutant lines and one heterozygous line of the mutant *CYP707A4* gene were used for the infection tests. Each plant of the heterozygous *cyp707A4* mutant line was verified by PCR prior to infection to confirm the mutation in the heterozygous state and to exclude a potential wild type character.

### 4.3. Endogenous ABA Concentration

Mutants and wild type (WT) plants were grown under hydroponic conditions on KNOP medium under a 12 h day/12 h night photoperiod (125 µmol m^−2^ s^−1^) at 21 °C. Leaves were collected from 4-week-old plants. To estimate ABA concentration, an Abscisic acid ELISA quantitation kit (Agrisera, Vännäs, Sweden) was used according to the manufacturer’s manual with some modifications during sample preparation. Fresh plant material (ca. 0.1–0.2 g FW of leaf) was collected and homogenized in liquid nitrogen, and next, vortexed overnight at 4–5 °C and 300 rpm in 0.9 mL of sample extraction buffer (Agrisera, Vännäs, Sweden). Samples were next centrifuged at 4 °C and 15,000 rpm for 15 min, and supernatans were used for analysis.

### 4.4. RNA Isolation and cDNA Synthesis

Infection sites of *H. schachtii* on Col-0 plants were hand-dissected at 2 and 4 dpi and immediately frozen in liquid nitrogen. Three biological replicates were collected, each containing ~50 mg of fresh root weight (≥100 infection sites per replicate). As a control, uninfected roots without root tips were collected.

Total RNA was isolated using a GeneMATRIX Universal RNA Purification Kit (EURx, Gdansk, Poland) with an additional step of on-column DNase I treatment. RNA concentration, purity and integrity were tested by spectrophotometric method with a BioSpectrometer^®^ equipped with a µCuvette (Eppendorf, Hamburg, Germany). RNA was electrophoretically separated in 1% (*w*/*v*) agarose gels in 1x TBE running buffer, visualized by SimplySafe (EURx) and photographed. After equalization of RNA concentrations, cDNA was synthesised using a High-Capacity cDNA Reverse Transcription Kit with the MultiScribe Reverse Transcriptase and random primers (Thermo Fisher Scientific, Waltham, MA, USA) according to the manufacturer’s instructions. The *UBP22* was used as a reference gene [28]. Primers are listed in Table 2.

### 4.5. Quantitative Real-Time PCR

Real-time qPCR reactions were performed in 96-well plates using a CFX Connect Real-Time PCR Detection System (Bio-Rad, Hercules, CA, USA) according to the manufacturer’s instruction. Five µL of 1:25 diluted first-strand cDNA was used as a template in qPCR. Apart from cDNA, each reaction contained 7.5 µL of iTaq Universal SYBR Green Supermix (Bio-Rad), 0.3 µL of each primer (final concentration 0.2 µM) and 1.9 µL of sterile water. qPCR conditions were as follows: polymerase activation at 95 °C for 2 min, then 40 cycles consisting of denaturation at 95 °C for 15 s and annealing/extension at 60 °C for 60 s with fluorescence reading. The specificity of amplified PCR products was verified by melting curve analysis.

### 4.6. Statistical Analysis

For gene expression analysis, real-time PCR was performed on three biological and two technical replicates. The calculation of reaction efficiency was performed using LinRegPCR software [29]. ABA measurements were performed in four biological repeats. The nematode infection assay was performed with at least nine biological replicates for genotype (n > 45). Significance of the data was calculated at *p* < 0.05 by performing Fisher’s multiple range test using one-way ANOVA and least significant difference (LSD) tests.

## Figures and Tables

**Table 1:** *A. thaliana* mutants used in this study.

Gene Name	Gene Acc. No.	Mutant NASC Stock No./Polymorphism	Gene Product	Gene Function	Mutant Phenotype
*ABI1* (*ABA-insensitive1*)	AT4G26080	SALK_072009 /abi1-2	2C protein phosphatase	ABA signalling	[23]
*ABI2* (*ABA-insensitive2*)	AT5G57050	SAIL_547_C10	2C protein phosphatase		nr *
*ABI5* (*ABA-insensitive5*)	AT2G36270	SALK_013163 /abi5-2	transcription factor		[24]
*PYL5* (*PYR1-like5*)	AT5G05440	SM_3_532	Receptor		nr *
*PYL6* (*PYR1-like6*)	AT2G40330	SAIL_1179_D01	Receptor		[25]
*CYP707A1*	AT4G19230	SALK_069127 /CYP707A1-1	cytochrome P450 monooxygenase	ABA catabolism	[26]
*CYP707A4*	AT3G19270	SALK_201208C	cytochrome P450 monooxygenase		nr *

* nr—no reference.

**Table 2:** Primer sequences used in this study.

Gene Name	Gene Acc. No.	Primers 5′-3′	Used for
*ABI1*	AT4G26080	TGAATATAGGAAGTCTGAAGCAAGTG CGAAACAGCATCTTCCATCTC	Genotyping
		ATGTCGAGATCCATTGGCGAT TTGCCATCTCACACGCTTCT	Real-time PCR
*ABI2*	AT5G57050	TTCCTTCTCCTCTTTTCTCCG TTGATCCGAGATCGATGAATC	Genotyping
		CGACTCTAGGGCGGTTTTGT AGGTATCTATCGCCAATGGATCT	Real-time PCR
*ABI5*	AT2G36270	CAATGGAAGTTCGGAATCATG CACTCGTTTTCTTCTTAAAGCG	Genotyping
		AGTGGATGGTCCAGTGGAGA CAACTCCGCCAATGCATGTT	Real-time PCR
*CYP707A1*	AT4G19230	CATGAACGTATTGGGTTTTGG TCCTGATATTGAATCCATCGC	Genotyping
		AGAAGCTGTCGAAGATGTCGAA GGGTTTTGGAGCCACCTCG	Real-time PCR
*CYP707A4*	AT3G19270	TCGATCATTTACAACATAAG GGAAGACCGTTTTGAGGTAT	Genotyping
		AAGGTCGTGTGCTAACCCAA AGCCTTTTGCTCAGCCTTAAC	Real-time PCR
*PYL5*	AT5G05440	TGGACACAAGGATCAACCAAC CAGCGAACAAGATCAAAAAGC	Genotyping
		CCGTGGTGGTGGAGTCTTAC AGAGACTGAAGGTTGCACCG	Real-time PCR
*PYL6*	AT2G40330	GCCTCGAGACAGTAGAAGATTG CGTATGACTCAACGACACGTG	Genotyping
		TATCGGAGACGGTCGAGAGG ACCAACGACGCTGAAACTGA	Real-time PCR
*UBP22*	AT5G10790	CACAAGGGGATGTTGGAATCAG ACTCACATCCTCTCACCACTTC	Real-time PCR

## Data Availability

The datasets used and/or analysed during the current study are available from the corresponding author on reasonable request.

## References

[1] Decraemer W., Hunt D.J., Perry R.N., Moens M. (2006). Structure and classification. Plant Nematology.

[2] Nicol J.M., Turner S.J., Coyne D.L., den Nijs L., Hockland S., Maafi Z.T., Jones J.T., Gheysen G., Fenoll C. (2011). Current nematode threats to world agriculture. Genomics and Molecular Genetics of Plant–Nematode Interactions.

[3] Sobczak M., Golinowski W., Jones J.T., Gheysen G., Fenoll C. (2011). Cyst nematodes and syncytia. Genomics and Molecular Genetics of Plant-Nematode Interactions.

[4] Jones J.T., Haegeman A., Danchin E.G.J., Gaur H.S., Helder J., Jones M.G.K., Kikuchi T., Manzanilla-López R., Palomares-Rius J.E., Wesemael W.M.L. (2013). Top 10 plant-parasitic nematodes in molecular plant pathology. Mol. Plant Pathol..

[5] Golinowski W., Grundler F.M.W., Sobczak M. (1996). Changes in the structure of Arabidopsis thaliana during female development of the plant-parasitic nematode Heterodera schachtii. Protoplasma.

[6] Haegeman A., Mantelin S., Jones J.T., Gheysen G. (2012). Functional roles of effectors of plant-parasitic nematodes. Gene.

[7] Glazebrook J. (2005). Contrasting Mechanisms of Defense Against Biotrophic and Necrotrophic Pathogens. Annu. Rev. Phytopathol..

[8] Ton J., Flors V., Mauch-Mani B. (2009). The multifaceted role of ABA in disease resistance. Trends Plant Sci..

[9] Cao F.Y., Yoshioka K., Desveaux D. (2011). The roles of ABA in plant-pathogen interactions. J. Plant Res..

[10] Bittner F., Oreb M., Mendel R.R. (2001). ABA3 Is a Molybdenum Cofactor Sulfurase Required for Activation of Aldehyde Oxidase and Xanthine Dehydrogenase in Arabidopsis thaliana. J. Biol. Chem..

[11] Xiong L., Zhu J.-K. (2003). Update on abscisic acid biosynthesis. Plant Physiol..

[12] Ng L.M., Melcher K., Teh B.T., Xu H.E. (2014). Abscisic acid perception and signaling: Structural mechanisms and applications. Acta Pharmacol. Sin..

[13] Weng J.K., Ye M., Li B., Noel J.P. (2016). Co-evolution of Hormone Metabolism and Signaling Networks Expands Plant Adaptive Plasticity. Cell.

[14] Nahar K., Kyndt T., Nzogela Y.B., Gheysen G. (2012). Abscisic acid interacts antagonistically with classical defense pathways in rice-migratory nematode interaction. New Phytol..

[15] Karimi M., Van Montagu M., Gheysen G. (1995). Exogenous application of abscisic acid to potato plants suppresses reproduction of Meloidogyne incognita. Mededelingen van de Faculteit Landbouwkundige en Toegepaste Biologische Wetenschappen.

[16] Sijmons P.C., Grundler F.M.W., Mende N., Burrows P.R., Wyss U. (1991). Arabidopsis thaliana as a new model host for plant-parasitic nematodes. Plant J..

[17] Kammerhofer N., Radakovic Z., Regis J.M.A., Dobrev P., Vankova R., Grundler F.M.W., Siddique S., Hofmann J., Wieczorek K. (2015). Role of stress-related hormones in plant defence during early infection of the cyst nematode Heterodera schachtii in Arabidopsis. New Phytol..

[18] Fuller V.L., Lilley C.J., Urwin P.E. (2008). Nematode resistance. New Phytol..

[19] Van Schie C.C.N., Takken F.L.W. (2014). Susceptibility Genes 101: How to Be a Good Host. Annu. Rev. Phytopathol..

[20] Gheysen G., Mitchum M.G. (2019). Phytoparasitic nematode control of plant hormone pathways. Plant Physiol..

[21] Szakasits D., Heinen P., Wieczorek K., Hofmann J., Wagner F., Kreil D.P., Sykacek P., Grundler F.M.W., Bohlmann H. (2009). The transcriptome of syncytia induced by the cyst nematode Heterodera schachtii in Arabidopsis roots. Plant J..

[22] Okamoto M., Kushiro T., Jikumaru Y., Abrams S.R., Kamiya Y., Seki M., Nambara E. (2011). ABA 9′-hydroxylation is catalyzed by CYP707A in Arabidopsis. Phytochemistry.

[23] Saez A., Robert N., Maktabi M.H., Schroeder J.I., Serrano R., Rodriguez P.L. (2006). Enhancement of Abscisic Acid Sensitivity and Reduction of Water Consumption in Arabidopsis by Combined Inactivation of the Protein Phosphatases Type 2C ABI1 and HAB1. Plant Physiol..

[24] Guo C., Jiang Y., Shi M., Wu X., Wu G. (2021). ABI5 acts downstream of miR159 to delay vegetative phase change in Arabidopsis. New Phytol..

[25] Antoni R., Gonzalez-Guzman M., Rodriguez L., Peirats-Llobet M., Pizzio G.A., Fernandez M.A., De Winne N., De Jaeger G., Dietrich D., Bennett M.J. (2013). PYRABACTIN RESISTANCE1-LIKE8 Plays an Important Role for the Regulation of Abscisic Acid Signaling in Root. Plant Physiol..

[26] Okamoto M., Kuwahara A., Seo M., Kushiro T., Asami T., Hirai N., Kamiya Y., Koshiba T., Nambara E. (2006). CYP707A1 and CYP707A2, Which Encode Abscisic Acid 8′-Hydroxylases, Are Indispensable for Proper Control of Seed Dormancy and Germination in Arabidopsis. Plant Physiol..

[27] Wiśniewska A., Wojszko K., Różańska E., Lenarczyk K., Kuczerski K., Sobczak M. (2021). Arabidopsis thaliana Myb59 Gene Is Involved in the Response to Heterodera schachtii Infestation, and Its Overexpression Disturbs Regular Development of Nematode-Induced Syncytia. Int. J. Mol. Sci..

[28] Wiśniewska A., Wojszko K., Różańska E., Lenarczyk K., Sobczak M. (2022). Arabidopsis thaliana AtHRS1 gene is involved in the response to Heterodera schachtii infection and its overexpression hampers development of syncytia and involves a jasmonic acid-dependent mechanism. J. Plant Physiol..

[29] Ramakers C., Ruijter J.M., Lekanne Deprez R.H., Moorman A.F.M. (2003). Assumption-free analysis of quantitative real-time polymerase chain reaction (PCR) data. Neurosci. Lett..

